# Cost-effectiveness analysis of neoadjuvant docetaxel plus androgen deprivation therapy before radical prostatectomy in high-risk localized prostate cancer

**DOI:** 10.3389/fpubh.2026.1786699

**Published:** 2026-05-05

**Authors:** Qi Liu, Yu Wang, Wenling Yuan, Junru Chen, Fangyuan Tian, Mei Zhan, Mengran Guo

**Affiliations:** 1 Department of Pharmacy, West China Hospital, Sichuan University, Chengdu, China; 2 Department of Urology, Institute of Urology, West China Hospital, Sichuan University, Chengdu, China

**Keywords:** androgen deprivation therapy, cost-effectiveness analysis, docetaxel, neoadjuvant therapy, prostate cancer

## Abstract

**Objective:**

High-risk localized prostate cancer is often associated with a higher risk of disease progression post-surgery. While radical prostatectomy (RP) is a cornerstone treatment, neoadjuvant chemohormonal therapy (CHT) combining docetaxel plus androgen deprivation therapy (ADT) prior to RP has shown promising efficacy in improving survival outcomes. This study aimed to evaluate the economic efficacy of neoadjuvant CHT followed by RP based on the results of CALGB 90203 from the perspective of Chinese healthcare system.

**Methods:**

A partitioned survival model with three states: event-free survival (EFS), progressive disease (PD), and death, was constructed. Clinical efficacy data, including overall survival and event-free survival, were obtained from the CALGB 90203 trial. Utility values, costs of drugs, surgery, follow-up, and adverse events were derived from local data and literature. Lifetime costs, quality-adjusted life-years (QALYs), and the incremental cost-effectiveness ratio (ICER) of neoadjuvant therapy vs. surgery alone per QALY gained were calculated, with a 5% annual discount rate applied. Cost-effectiveness was assessed against a willingness-to-pay (WTP) threshold of $41,859 per QALY. One-way and probabilistic sensitivity analyses were conducted to test model robustness.

**Results:**

Compared with RP alone, the neoadjuvant CHT strategy provided an additional 1.08 QALYs at an incremental cost of $5,663, resulting in an ICER of $5,238 per QALY. The model was most sensitive to changes in health utility values of EFS and PD state. Probabilistic sensitivity analysis indicated a 96.40% probability that neoadjuvant CHT is cost-effective at the defined threshold.

**Conclusion:**

From the Chinese payer's perspective, neoadjuvant CHT prior to radical prostatectomy is a highly cost-effective treatment strategy for patients with high-risk localized prostate cancer even when accounting for the upfront costs and quality-of-life impact associated with treatment-related adverse events.

## 1 Introduction

Prostate cancer represents a frequently encountered malignancy among men worldwide (1). In China, both the incidence and mortality rates have been rising steadily, posing a growing challenge to the healthcare system (2, 3). Radical prostatectomy (RP) is a standard curative-intent treatment for localized prostate cancer. However, patients with high-risk features–defined by elevated PSA, high Gleason score (4–6), or advanced clinical stage (T3a) — remain at substantially increased risk of biochemical recurrence, metastasis, and prostate cancer-specific mortality even after successful surgery (7). This persistent postoperative vulnerability represents a critical unmet clinical need: these patients require more effective perioperative systemic strategies to eradicate micrometastases, reduce local recurrence, and ultimately improve long-term survival. This unmet need has spurred investigation of multimodal strategies. Therefore, the strategy of employing neoadjuvant therapy before RP surgery to reduce tumor burden, eradicate micrometastases, and improve surgical outcomes has been developed for these high-risk patients. Given the limited efficacy of neoadjuvant androgen-deprivation therapy (ADT) alone – which has demonstrated histological benefits but no improvement in biochemical progression-free survival in multiple studies — intensifying treatment by incorporating docetaxel, a cornerstone treatment of metastatic prostate cancer with proven survival benefit, represents a promising strategy for high-risk localized prostate cancer (4, 5, 8–10). The clinical significance of this approach lies in its potential to convert a high-risk, biologically aggressive cancer into a more favorable postsurgical prognosis, thereby offering patients a chance for prolonged disease-free and overall survival beyond what RP alone can achieve.

The pivotal phase III CALGB 90203 trial, conducted across multiple institutions in the United States, evaluated this strategy of neoadjuvant chemohormonal therapy (CHT) combining docetaxel plus ADT followed by RP, vs. RP alone. In this multicenter randomized controlled trial, 788 men with clinically localized high-risk prostate cancer were assigned in a 1:1 ratio to either neoadjuvant CHT (six cycles of docetaxel 75 mg/m^2^ every 3 weeks plus ADT for 18–24 weeks) followed by RP, or RP alone. The primary endpoint was 3-year biochemical progression-free survival (BPFS), defined as freedom from biochemical failure, where biochemical failure was defined as serum PSA >0.2 ng/ml on two consecutive occasions at least 3 months apart. Secondary endpoints included 5-year BPFS, overall BPFS, metastasis-free survival (MFS), prostate cancer-specific mortality, and overall survival (OS). While the primary biochemical endpoint was confounded by the early and frequent use of salvage therapy, the trial demonstrated significant and clinically meaningful improvements in overall survival (HR, 0.61) and metastasis-free survival (HR, 0.70) (6). Despite these benefits, the regimen adds substantial upfront costs and management complexity related to drug use and adverse events (6, 11, 12). In an era of escalating healthcare costs and emphasis on value-based care, application of such intensive therapy should be justified by both clinical efficacy and economic value (13, 14).

Several other clinical trials have investigated neoadjuvant strategies for high-risk localized prostate cancer, including novel androgen receptor pathway inhibitors such as enzalutamide and abiraterone combined with ADT (15–17). These phase II studies have shown encouraging pathologic responses. However, most of these trials lack long-term survival outcome data, and no phase III trial has yet demonstrated a survival benefit comparable to that observed with docetaxel-based CHT. Given that CALGB 90203 remains the only phase III randomized controlled trial providing mature survival data (median follow-up 6.1 years, up to 12.1 years) with confirmed OS and MFS improvements for neoadjuvant docetaxel plus ADT, it serves as the most robust evidence base for economic evaluation. Moreover, the availability of detailed event-free survival and adverse event profiles from this trial enables a comprehensive cost-effectiveness analysis from a long-term perspective. Although the CALGB 90203 trial was conducted in a predominantly White US population, no equivalent phase III trial with mature survival data exists in Chinese patients with high-risk localized prostate cancer; therefore, these data represent the best available evidence, and the relative treatment effect is generally considered transferable across populations when baseline risk is appropriately calibrated.

Therefore, the economic assessment of this regimen is particularly critical within the context of China's rapidly evolving healthcare landscape. The nation is experiencing demographic aging, which fuels a continuous rise in prostate cancer incidence. Concurrently, profound payment reforms are underway, including the implementation of Diagnosis-Intervention Packet (DIP) and Diagnosis-Related Group (DRG) systems, alongside annual National Reimbursement Drug List (NRDL) negotiations (18, 19). These reforms aim to control expenditures while ensuring access to valuable innovations, making a formal cost-effectiveness analysis essential to determine whether the long-term survival benefits of neoadjuvant CHT justify its additional costs compared to RP alone.

Using clinical efficacy data from the CALGB 90203 trial and incorporating local Chinese cost data, this study aims to quantify the economic value of CHT prior to RP surgery in high-risk localized prostate cancer patients from the Chinese healthcare perspective, providing feasible evidence for decision-makers.

## 2 Methods

### 2.1 Patient information and intervention

For the base case analysis, we constructed a hypothetical cohort whose demographic and clinical characteristics were aligned with the patient population of the CALGB 90203 trial. The study included 788 men with clinically localized, high-risk prostate cancer, randomly assigned in a 1:1 ratio to receive either neoadjuvant chemohormonal therapy (CHT) followed by radical prostatectomy (RP) or RP alone. Patients in this economic evaluation were assumed to be consistent with the clinical trial population. All patients had histologically confirmed prostatic adenocarcinoma with clinical T1–3a disease, serum PSA ≤ 100 ng/ml, and no evidence of metastatic disease. Patients were considered high-risk if they met either of the following criteria: a Kattan nomogram–predicted probability of < 60% for 5–year biochemical progression–free survival after RP, or a biopsy Gleason score of 8–10 (20, 21). Patients randomly assigned to the neoadjuvant arm received docetaxel (75 mg/m^2^ every 3 weeks for 6 cycles) combined with androgen-deprivation therapy for 18–24 weeks prior to RP surgery. The control arm underwent immediate RP alone. Overall survival and event-free survival from the trial population were used to inform the long-term economic evaluation.

Notably, 3–year biochemical progression-free survival (BPFS), the primary clinical endpoint in the CALGB 90203 trial was compromised by early and frequent use of salvage therapies (48% of patients), which reduced events for this endpoint. However, the trial demonstrated robust improvements in overall survival (HR, 0.61) and metastasis-free survival (HR, 0.70), which form the clinical efficacy foundation for our economic evaluation. These endpoints were less affected by salvage therapy interventions and represent more meaningful clinical outcomes for patients.

### 2.2 Model structure

**Figure 1:**
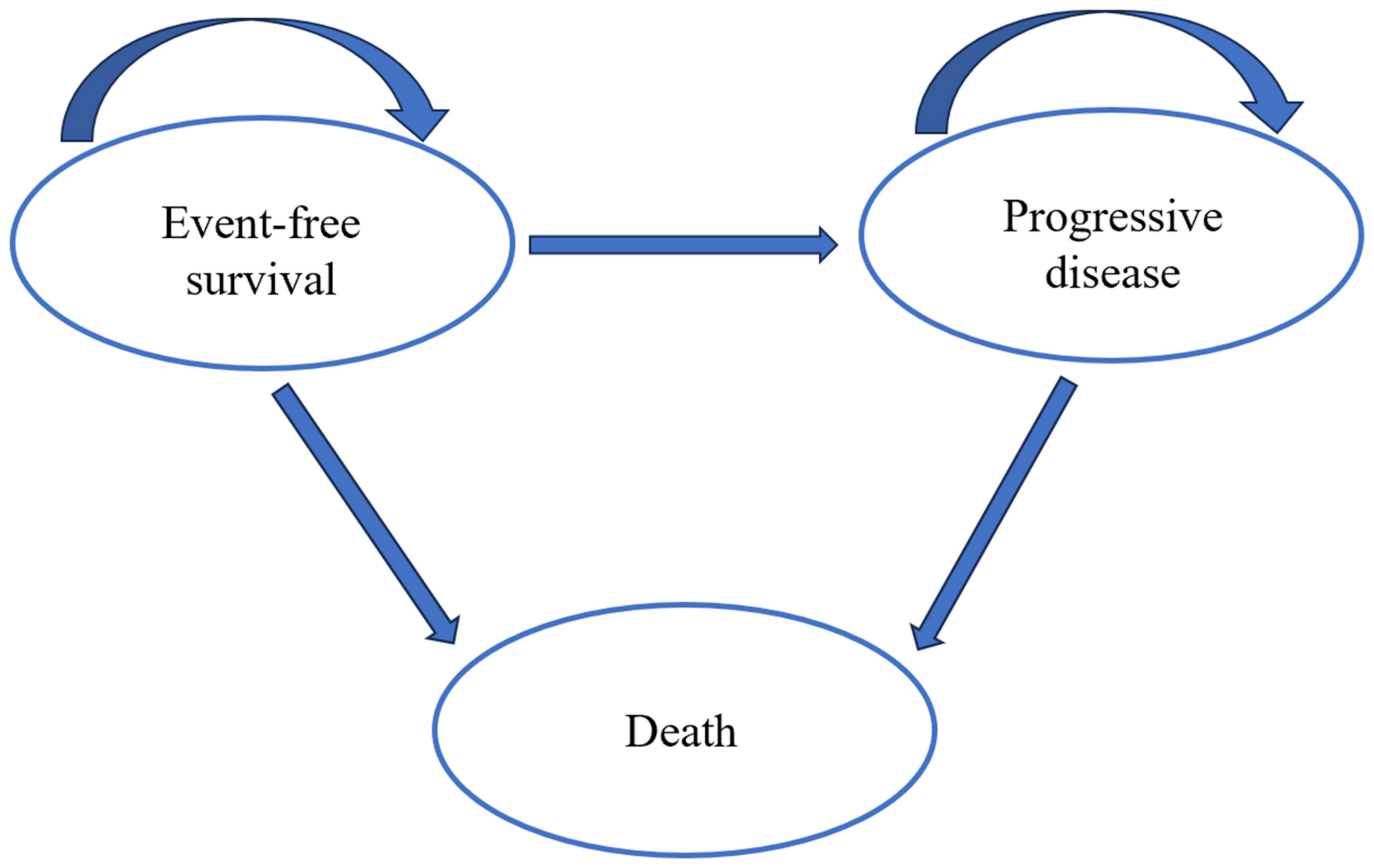
The three health states of partitioned survival model.

We constructed a partitioned survival model with TreeAge Pro 2020 (Treeage Software, Inc., MA, USA), comprising three mutually exclusive health states: Event-Free Survival (EFS), Progressive Disease (PD), and Death. All patients entered the model in the EFS state and could transition to PD or Death in each monthly cycle (Figure 1). The PD state was explicitly defined as biochemical recurrence according to the criteria of CALGB 90203 trial (PSA > 0.2 ng/ml with a subsequent rise). It is worth noting that a significant proportion of patients in the trial (43% in the neoadjuvant CHT arm and 52% in the RP-alone arm) received salvage therapy (radiotherapy ± ADT) before reaching PSA-based endpoint. To ensure consistency with the reported intention-to-treat efficacy data (EFS and OS curves) from the trial, the model utilized this formal endpoint to define the transition from the EFS to the PD state. However, to accurately reflect real-world clinical management and associated resource use, we have introduced a comprehensive monthly cost for the PD state. This cost consists of costs for ongoing monitoring, salvage radiotherapy, and subsequent systemic therapies that would be initiated upon signs of recurrence—whether identified strictly by the formal PSA criterion or by earlier clinical discretion prompting intervention. In summary, the model distinguishes between the definition of clinical states (based on trial protocol) and the assignment of costs (based on real-world practice) to ensure both clinical consistency and economic relevance.

The transitions between health states were not governed by explicit probabilities (e.g., transition matrices). Instead, they were determined directly from the extrapolated EFS and OS survival curves. Specifically, the proportion of patients in the EFS state at any time t was defined by the value of the EFS curve at time t. The proportion of patients who had died was defined as 1 minus the value of the OS curve at time t. The proportion of patients in the PD state (alive with progression) was then calculated as the difference between the OS and EFS curve values at time t. EFS was defined according to the CALGB 90203 trial protocol as the time from randomization to the first occurrence of any of the following events: biochemical progression (PSA > 0.2 ng/ml with two consecutive rises measured at least 3 months apart), initiation of subsequent androgen-deprivation therapy, receipt of radiation therapy more than 6 months after RP, local or distant progression, or death from any cause. This composite endpoint captures clinically meaningful treatment failure. It is also less susceptible to the informative censoring that compromised the trial's original BPFS endpoint.

The model was parameterized with a 20–year time horizon and a 1-month cycle length, aligning with the administration pattern of ADT (22). For reproducibility of the TreeAge Pro 2020 analysis, the following settings and modeling assumptions were applied: a cohort simulation type using a half-cycle correction to account for the continuous-time nature of events; the calculation method was set to “Expected Value” with a convergence tolerance of 0.001; all survival curves were entered as time-dependent probabilities using the parametric distribution functions (Weibull, Gamma, or Gen Gamma) with their respective shape and scale parameters estimated from the CALGB 90203 trial data; the “State Residence Time” method was used to track the proportion of patients in each health state over time; no tunnel states or tracker variables were required. Costs and health outcomes were discounted at an annual rate of 5%, as recommended by guidelines, with a range of 0% to 8% tested in sensitivity analyses. Cost-effectiveness was assessed using the incremental cost-effectiveness ratio (ICER), calculated as the incremental cost per quality-adjusted life-year (QALY) gained. The willingness-to-pay (WTP) threshold was set at three times the Chinese GDP per capita (US$ 41,859 per QALY), consistent with WHO-CHOICE recommendations and widely used standards for cost-effectiveness evaluations in China. For enhanced policy relevance, results were also evaluated against a stricter threshold of one time GDP per capita (US$ 13,953 per QALY). An ICER below this threshold was considered cost-effective.

### 2.3 Clinical parameters

**Figure 2:**
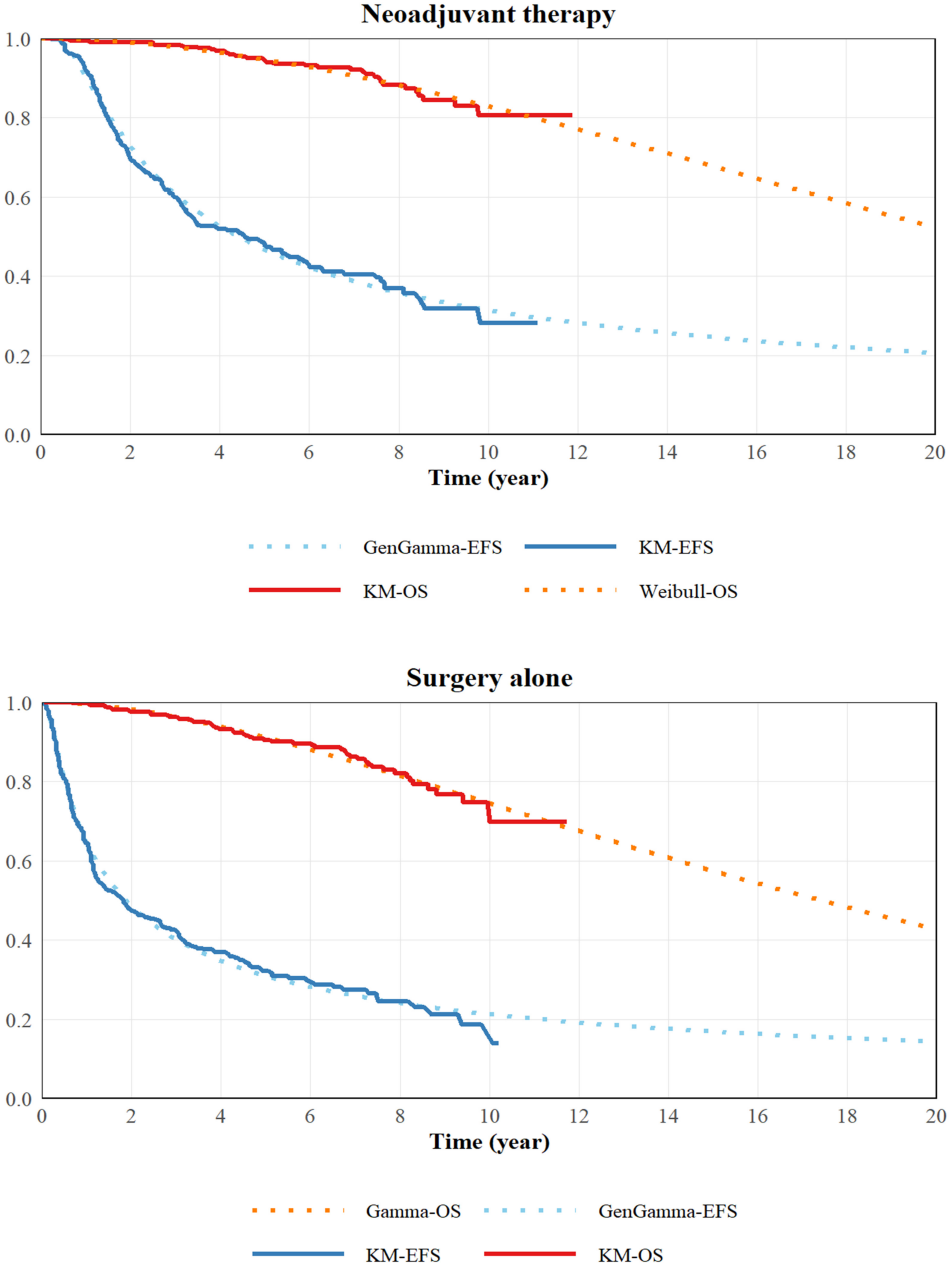
Kaplan Meier overall and event- free survival curves and the fitting curves for the CHT and RP groups.

Individual patient data for EFS and OS were reconstructed from the Kaplan-Meier survival curves reported in the clinical trial using the GetData Graph Digitizer 2.26 (Fedorov S, Russia). Parametric survival distributions, including Weibull, Exponential, Gompertz, Gamma, Log-logistic, and Log-normal, were fitted to the reconstructed data using R software. We selected the best-fitting parametric distributions for the base case based on the Akaike (AIC) and Bayesian (BIC) information criteria, guided by visual inspection of the survival curves. Based on the IC values (Table 1), the Weibull and Gamma distributions were selected as the best fits for the OS curves in the neoadjuvant therapy and surgery-alone groups, respectively. The Gen Gamma distribution provided the best fit for the EFS curves in both treatment groups. The original and fitting curves were displayed in Figure 2. Since utility values for health states were not available from the primary trial, to address this gap, they were derived from the published literature. The utility values assigned for the EFS, PD, and death health states were 0.844, 0.612, and 0, respectively (23).

**Table 1:** Goodness-of-fit to Kaplan-Meier survival curves from the CALGB 90203 trial.

Strategy	Neoadjuvant therapy				Surgery alone			
**Parametric distribution**	**OS**		**EFS**		**OS**		**EFS**	
	AIC	BIC	AIC	BIC	AIC	BIC	AIC	BIC
Exponential	378.08	382.04	1,195.39	1,199.36	498.00	501.99	1,205.09	1,209.08
Gamma	367.88	375.81	1,191.33	1,199.27	483.21	491.18	1,185.95	1,193.92
Gen Gamma	369.25	381.15	1,136.70	1,148.61	485.21	497.16	1,108.26	1,120.21
Gompertz	367.63	375.57	1,194.47	1,202.41	486.38	494.34	1,143.08	1,151.05
Weibull	367.41	375.35	1,195.01	1,202.95	483.25	491.33	1,174.96	1,182.93
Log-logistic	367.65	375.59	1,170.46	1,178.40	483.37	491.34	1,140.04	1,148.01
Log-normal	370.67	378.61	1,155.62	1,163.56	483.92	491.88	1,125.50	1,133.46

### 2.4 Cost estimation

The study only incorporates the direct medical costs related to the treatment plan, which included drug costs, surgery fees, costs for managing adverse events (AEs), follow-up, diagnostic examinations, and best supportive care (BSC). Drug dosages were calculated based on a standard body surface area of 1.72 m^2^ and a standard male body weight of 65 kg. The prices for drugs and examinations were sourced from Sichuan Provincial Drug and Medical Device Bidding and Procurement Service Center and West China Hospital (2025 charge standard). We converted all costs to US dollars using the average exchange rate for 2025 of $1 = ¥6.8974. AE cost calculations were based on the incidence rates of grade 3/4 adverse events reported in the CALGB 90203 trial, more details were shown Table 2. The model incorporated costs for subsequent therapies informed by the CALGB 90203 trial. Specifically, the trial data showed that 13% and 23% of patients in the neoadjuvant CHT and RP alone arms, respectively, received adjuvant radiotherapy combined with ADT. The cost of this regimen was included for the corresponding proportion of patients in each arm. Furthermore, the significant improvement in metastasis-free survival with neoadjuvant CHT translates into substantial long-term cost savings, as fewer patients progress to require lifelong, expensive treatments for metastatic disease. These savings are captured in the model through reduced time and associated costs in the progressive disease (PD) health state.

**Table 2:** Base-case values.

Parameters	Base	Distribution	Source
Costs ($)			
Docetaxel (Taxotere^®^) per 20mg	131.93	Gamma	Yaozh database
Dexamethasone per 75mg	0.67	Gamma	Yaozh database
Leuprolide (ENANTONE^®^) per 3.75mg	195.97	Gamma	Yaozh database
Radical prostatectomy surgery	8,228.46	Gamma	West China Hospital
Radiotherapy	7,249.11	Gamma	West China Hospital
PSA	13.34	Gamma	West China Hospital
Laboratory test	17.25	Gamma	West China Hospital
CT	150.06	Gamma	West China Hospital
Bone scan	91.34	Gamma	West China Hospital
Follow-up per cycle	52.03	Gamma	Reference (34)
Best supportive care per cycle	118.31	Gamma	Reference (34)
Adverse Event Cost ($)			
Hyperglycemia	16.50	Gamma	Reference (35)
Neutropenia	470.80	Gamma	Reference (34)
Febrile neutropenia	962.81	Gamma	Reference (34)
Fatigue	109.85	Gamma	Reference (34)
Risk of adverse events in CHT group (%)			
Hyperglycemia	6%	Beta	Reference (6)
Neutropenia	23%	Beta	Reference (6)
Febrile neutropenia	4%	Beta	Reference (6)
Fatigue	5%	Beta	Reference (6)
Utility			
EFS	0.844	Beta	Reference (23)
PD	0.612	Beta	Reference (23)
Disutility			
Hyperglycemia	−0.040	Beta	Reference (24)
Neutropenia	−0.200	Beta	Reference (36)
Febrile neutropenia			
Fatigue			
Febrile neutropenia	−0.416	Beta	Reference (36)
Fatigue			
Fatigue	−0.068	Beta	Reference (35)

PSA, prostate specific antigen; CT, computed tomography; EFS, event-free survival; PD, progressive disease.

### 2.5 Sensitivity analysis

We performed a one-way sensitivity analysis to evaluate the impact of parameter uncertainty on the ICER. For key parameters, ranges were derived from explicit sources: health utility values for event-free survival (EFS) and progressive disease (PD) were based on the 95% confidence intervals reported; the cost of docetaxel was varied between the National Volume-Based Procurement (NVBP) price and the originator price; the cost of radical prostatectomy was varied based on the interquartile range of local hospital billing data. For parameters without published confidence intervals or price variation data (e.g., laboratory test costs, follow-up costs), values were modified by ± 20% of the base-case value. The discount rate was varied from 0% to 8% as recommended by Chinese guidelines. To further evaluate model uncertainty, we conducted a probabilistic sensitivity analysis (PSA) involving 1,000 Monte Carlo simulations. Costs and utilities were assigned Gamma and Beta distributions, respectively. Specifically, for each simulation, parameter values were drawn from their respective distributions using second-order Monte Carlo sampling with 1,000 iterations. The random number generator was set to the default Mersenne Twister algorithm. PSA results are presented as a cost-effectiveness acceptability curve and a scatter plot of incremental costs and effects.

We also conducted several scenario analyses to test the robustness of our base–case results under alternative key assumptions. First, the time horizon was shortened from 20 years to 10 years to assess the impact of long-term survival extrapolation. Second, the price of docetaxel was reduced from the base-case value (US$ 131.93 per 20 mg) to the National Volume-Based Procurement (NVBP) price (US$ 3.14 per 20 mg) to reflect current real-world acquisition costs in China. Third, the willingness-to-pay threshold was lowered from 3 × GDP per capita (US$ 41,859/QALY) to 1 × GDP per capita (US$ 13,953/QALY) to align with stricter health technology assessment criteria.

## 3 Results

### 3.1 Base-case analysis

Our base-case analysis showed that the total treatment cost for the neoadjuvant CHT plus RP strategy was $17,496, compared to $11,833 for RP alone, resulting in an incremental cost of $5,663 (Table 3). This modest net cost difference is a result of the significant offsetting of upfront expenses (drugs and AE management) by downstream savings in the neoadjuvant arm. Specifically, the neoadjuvant group requires less adjuvant radiotherapy plus ADT (13% vs. 23% in the RP alone arm) and incurs lower long-term costs due to a lower incidence of progressive disease. In terms of effectiveness, the neoadjuvant CHT plus RP strategy yielded 11.27 QALYs, compared to 9.35 QALYs for the RP-alone group, resulting in a gain of 1.08 QALYs. The calculated incremental cost-effectiveness ratio (ICER) was $5,238 per QALY gained. This figure is much lower than the preset WTP threshold of $41,859 per QALY, indicating that the neoadjuvant CHT strategy is cost-effective. At a threshold of 1 × GDP per capita (US$ 13,953/QALY), the ICER of US$ 5,238/QALY remained well below the threshold, confirming the strategy is highly cost-effective.

**Table 3:** Base-line results: cost-effectiveness analysis (20 years).

Strategy	Neoadjuvant therapy	Surgery
Cost ($)		
EFS State ($)	16,699	10,831
PD state ($)	797	1,002
Total cost ($)	17,496	11,833
Incremental costs ($)	5,663	–
Effectiveness (QALYs)		
EFS state (QALYs)	6.48	3.62
PD state (QALYs)	4.79	5.74
Total effectiveness (QALYs)	11.27	9.35
Incremental effectiveness (QALYs)	1.08	–
ICERs vs. surgery alone ($/QALY)	5,238	–

### 3.2 Sensitivity analysis

**Figure 3:**
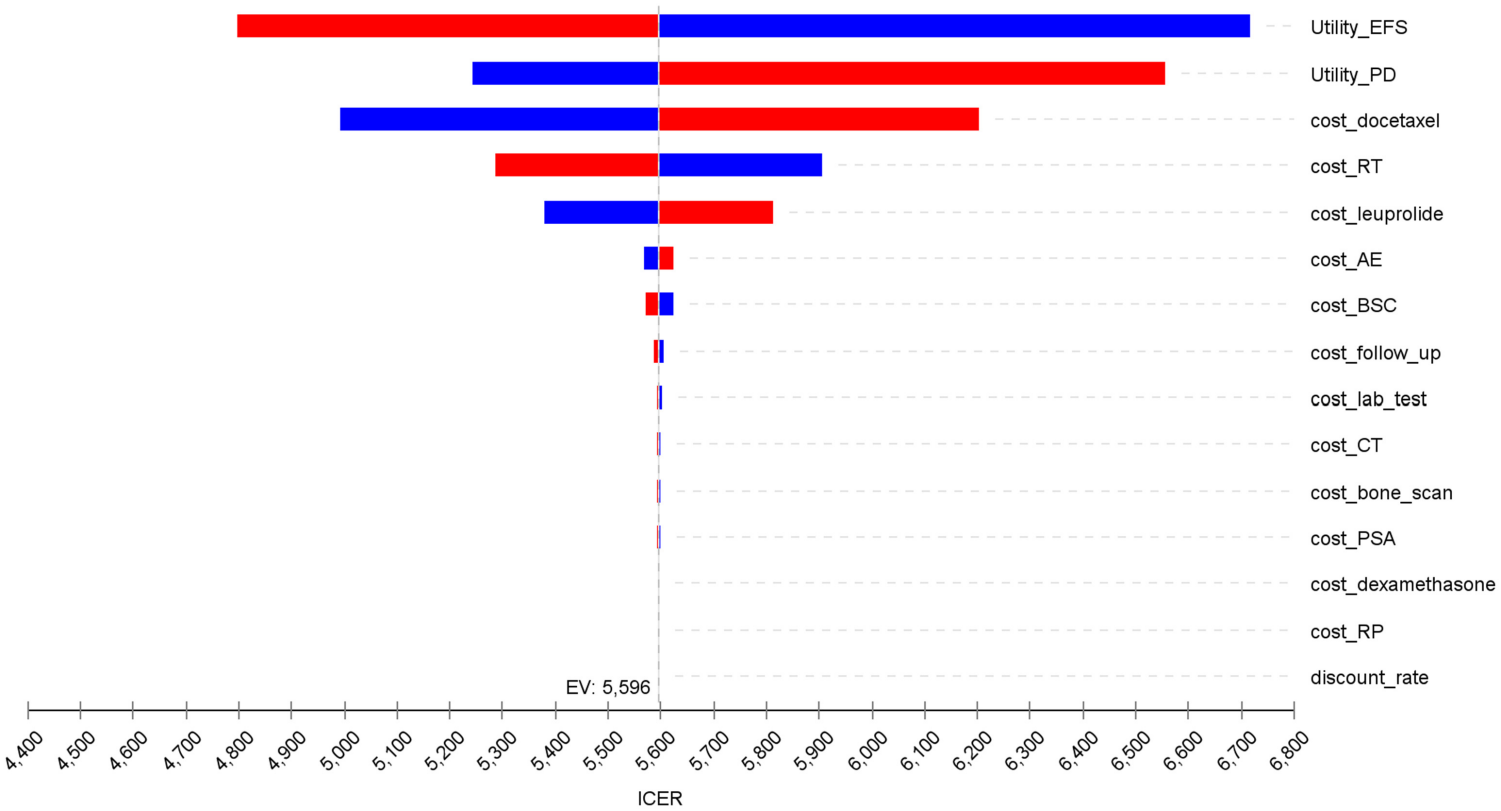
Tornado diagram summarizing the one-way sensitivity analysis results. Blue bars represent the ICER when the parameter is set to its lower bound, and red bars represent the ICER when the parameter is set to its upper bound. EFS, event-free survival; PD, progressive disease; RT, radiotherapy; AE, adverse event; RP, radical prostatectomy surgery; BSC, best supportive care; CT, computed tomography; PSA, prostate specific antigen.

**Figure 4:**
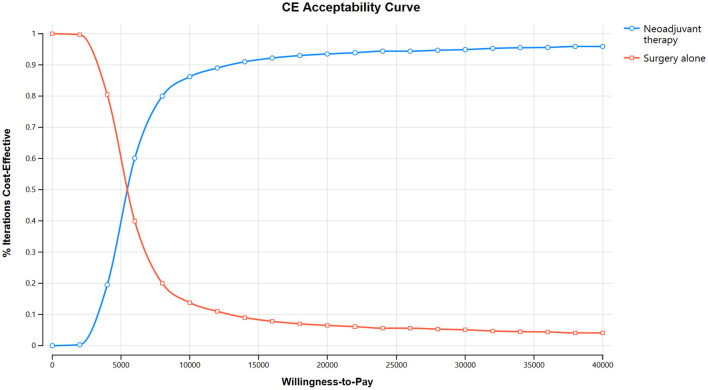
Cost-effectiveness acceptable curve (CEAC): neoadjuvant chemohormonal therapy vs. radical prostatectomy surgery alone.

**Figure 5:**
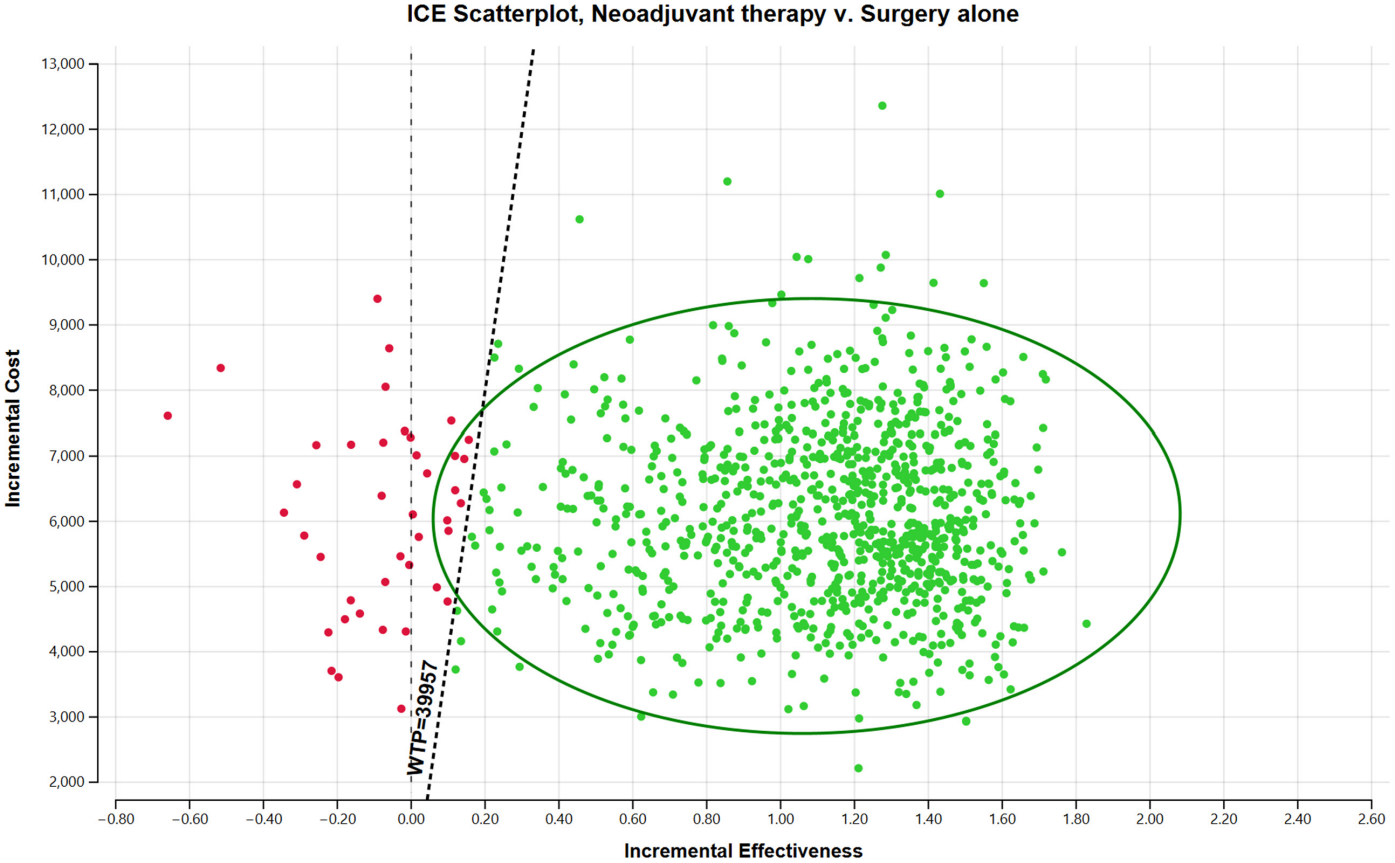
Scatter plot of ICE from probabilistic sensitivity analysis. **(A)** Willingness-to-pay (WTP) threshold = US$13,953 per QALY (1 × GDP per capita). **(B)** WTP threshold = US$41,859 per QALY (3 × GDP per capita). In each panel, green points represent simulations where the incremental cost-effectiveness ratio (ICER) is below the respective WTP threshold (cost-effective), and red points represent simulations where the ICER is above the threshold (not cost-effective).

The one-way sensitivity analysis demonstrated that the model was most sensitive to variations in the health utility values for event-free survival (EFS) and post-progression (PD) survival, followed by the costs of docetaxel and radical prostatectomy surgery (Figure 3). Additional influential parameters included the costs of leuprolide, AE management and BSC, reflecting the role of cost-offset mechanisms in the treatment pathway. Within all the tested reasonable ranges, the incremental ICER remained consistently between $4,100 and $6,400 per QALY gained, well below the predefined WTP threshold. The probability sensitivity analysis further confirmed this robustness: the cost-effectiveness acceptability curve (CEAC) demonstrated that the probability of neoadjuvant therapy being cost-effective increased with higher WTP thresholds (Figure 4). At the predefined threshold of $41,859 per QALY, neoadjuvant CHT regimen prior to RP surgery had a 96.40% probability of being cost-effective compared to surgery alone (Figure 5).

The results of the scenario analyses are summarized in Table 4. When the time horizon was shortened to 10 years (Scenario 1), the neoadjuvant CHT strategy yielded an additional 0.46 QALYs at an incremental cost of $5,642, resulting in an ICER of $12,157 per QALY—still well below the 3 × GDP willingness-to-pay (WTP) threshold ($41,859 per QALY). When the docetaxel price was reduced to the National Volume-Based Procurement (NVBP) level of $3.14 per 20 mg (Scenario 2), the incremental cost dropped to $679, and the ICER decreased to $628.14 per QALY, reflecting substantial cost savings from the real-world Chinese procurement policy. Finally, at the stricter WTP threshold of 1 × GDP per capita ($13,953 per QALY), the neoadjuvant CHT regimen remained cost-effective with a probability of 90.30% based on the probabilistic sensitivity analysis. Collectively, these scenario analyses confirm that the base-case conclusion—that neoadjuvant docetaxel plus ADT prior to radical prostatectomy is highly cost-effective for high-risk localized prostate cancer in China—is robust across alternative assumptions.

**Table 4:** Scenario analysis.

Strategy	Neoadjuvant therapy	Surgery
Scenario 1. Simulated life: 10 years		
Cost ($)	17,084	11,422
Effectiveness (QALYs)	5.55	5.09
Incremental costs ($)	5,642	–
Incremental effectiveness (QALYs)	0.46	–
ICERs vs. surgery alone ($/QALY)	12,156.96	–
Scenario 2. Docetaxel price: NVBP (US$ 3.14 per 20 mg)		
Cost ($)	12,512	11,833
Effectiveness (QALYs)	7.72	6.64
Incremental costs ($)	679	–
Incremental effectiveness (QALYs)	1.08	–
ICERs vs. surgery alone ($/QALY)	628.14	–

## 4 Discussion

This study conducted a crucial economic assessment of neoadjuvant CHT regimen prior to RP surgery in high-risk localized prostate cancer patients based on the results of the CALGB 90203 trial from the Chinese healthcare perspective. While the definitive phase III trial concluded that its primary endpoint (3–year BPFS) was not met and cautioned against the routine use of neoadjuvant chemohormonal therapy due to toxicity, our cost-effectiveness analysis reveals a different dimension of value. By quantitatively modeling the trial's unequivocal secondary benefits in overall survival and event-free survival while explicitly accounting for the disutility of grade 3/4 adverse events, we demonstrate that this regimen is highly cost-effective in China, with an incremental cost-effectiveness ratio of $5,238 per QALY. This finding underscores a pivotal principle in health technology assessment: a therapy may fail a protocol-defined intermediate endpoint yet deliver significant long-term survival gains that translate into an efficient allocation of resources, particularly when it mitigates the substantial clinical and economic burden of disease progression and subsequent therapy (24–26).

The divergence between the trial's primary clinical conclusion and our favorable economic evaluation is both explicable and insightful, hinging critically on endpoint selection and the high proportion of early salvage therapy. The trial's BPFS endpoint required a serum PSA > 0.2 ng/ml that increased on two consecutive occasions at least 3 months apart. Notably, the investigators highlighted a key interpretative challenge: 48% of patients (52% in the RP-alone arm; 43% in the neoadjuvant CHT arm) received additional treatment, primarily salvage radiotherapy, before meeting this strict biochemical criterion. This clinically appropriate, real-world practice effectively censored these patients in the BPFS analysis, diluting the observable difference in the primary endpoint. In contrast, the trial's event-free survival analysis, which considered the initiation of any subsequent therapy (e.g., salvage radiation, ADT) as an event, revealed a significant benefit for neoadjuvant CHT (weighted HR 0.61). This supports the conclusion that the intervention meaningfully delayed the need for further treatment, a finding our economic model directly incorporates. Furthermore, the comprehensive monthly cost assigned to the PD state encompasses monitoring, salvage radiotherapy, and subsequent systemic therapies. This approach captures the essence of the “cost-offset” effect: the upfront costs of docetaxel and ADT, along with toxicity management (grade 3/4 adverse events occurred in 45% of patients during chemotherapy), are counterbalanced over time by a substantial delay in costly salvage therapies and the associated delay or avoidance of terminal metastatic care (26, 27).

Therefore, our analysis adds a crucial layer of evidence for decision-makers in China. It argues against a blanket dismissal of neoadjuvant CHT based solely on the negative primary endpoint. Instead, it supports the selective and strategic consideration of this regimen within a value-based framework for well-informed patients with high-risk localized disease who are fit enough to tolerate the treatment burden. The significant improvement in pathological outcomes (lower rates of seminal vesicle invasion, positive lymph nodes, and positive surgical margins in the neoadjuvant arm) likely underpins the observed delay in treatment failure (EFS benefit), which our model translates into long-term economic value. For policymakers navigating volume-based procurement and National Reimbursement Drug List (NRDL) negotiations, our findings provide a robust economic rationale for considering conditional reimbursement or managed entry agreements, ensuring access is aligned with patients most likely to derive a net benefit. Notably, our supplementary analysis at the 1 × GDP per capita WTP threshold further strengthens this rationale, as the neoadjuvant CHT strategy remains highly cost-effective even under the stricter economic evaluation standard, which is more in line with the current healthcare resource allocation context in China.

We acknowledge that this study also has several limitations. Notably, the clinical efficacy inputs are derived from a predominantly Western patient population, and thus the real-world performance and safety profile in Chinese patients need to be monitored. While Chinese patients with high-risk localized prostate cancer present with older age and higher PSA levels compared with the CALGB 90203 population, the higher baseline risk would, if anything, increase the absolute benefit of neoadjuvant CHT, making our base-case conclusion conservative (28). Furthermore, health utility values, including those for adverse events, were sourced from the published literature as such data were not collected in CALGB 90203, introducing potential uncertainty (29). We recognize that directly measured utility values from Chinese patients receiving neoadjuvant docetaxel plus androgen deprivation therapy for high-risk localized prostate cancer are not currently available. This evidence gap is not unique to our study; it reflects a broader challenge in the field, as noted in recent Chinese pharmacoeconomic studies (30). To address this limitation, we incorporated the best available Asian-specific evidence, which reported a mean EQ-5D-5L utility score of 0.71 (31). Notably, this value falls within the range tested in our one-way sensitivity analysis, and our original sensitivity analysis demonstrated that the ICER remained below the willingness-to-pay threshold across this entire range. Another consideration is that the research was conducted from the healthcare system perspective and does not include indirect costs, which are relevant for a full societal appraisal (32). Finally, long-term survival extrapolation introduces uncertainty as with all model-based studies, although standard methodologies and sensitivity analyses were employed (33).

Overall, this pharmacoeconomic evaluation reframes the narrative around neoadjuvant docetaxel-based CHT for high-risk localized prostate cancer in China. The strategy's substantial survival and treatment-delaying benefits, when combined with its powerful cost-offset mechanism, yield a highly favorable ICER even after factoring in treatment toxicity, making it a cost-effective investment for the Chinese healthcare system. These findings provide a data-driven rationale for clinicians and patients to consider this regimen as a high-value option in shared decision-making and offers policymakers an evidence-based foundation for facilitating selective access under a value-based healthcare paradigm. The consistency of cost-effectiveness conclusions across 3 × and 1 × GDP per capita WTP thresholds further confirms the robustness and clinical applicability of our findings.

## Data Availability

The original contributions presented in the study are included in the article/supplementary material, further inquiries can be directed to the corresponding authors.
